# Occurrence of Oxyurid-Type Nematode Eggs in Aldabra Giant Tortoises (*Aldabrachelys gigantea*) from Zoological Collections in Zanzibar, Tanzania

**DOI:** 10.3390/ani16162626

**Published:** 2026-08-21

**Authors:** Rusko Petrov, Blagovesta Slavcheva, Iskren Stanilov

**Affiliations:** 1 Department of General Animal Husbandry, Section Ecology and Zoology, Faculty of Veterinary Medicine, Trakia University, 6000 Stara Zagora, Bulgaria; rpetrov@greenbalkans.org; 2 Green Balkans—Stara Zagora NGO, 26 Konstantin Irechek Str, 6000 Stara Zagora, Bulgaria; 3 Unit of Parasitology and Parasitic Diseases, Department of Veterinary Microbiology, Infectious and Parasitic Diseases, Faculty of Veterinary Medicine, Trakia University, 6000 Stara Zagora, Bulgaria; 4 Department of Hygiene, Epidemiology, Microbiology, Parasitology and Infectious Diseases, Medical Faculty, Trakia University, 6000 Stara Zagora, Bulgaria; iskren.stanilov@trakia-uni.bg

**Keywords:** *Aldabrachelys gigantea*, Aldabra giant tortoises, oxyurid-type nematode eggs, coprological examination, Fülleborn’s method, sedimentation method, parasitology, Zanzibar, Tanzania

## Abstract

A parasitological survey was conducted during May 2026 on captive Aldabra giant tortoises (*Aldabrachelys gigantea*) housed at three zoological facilities on Zanzibar Island, Tanzania: the Prison Island Giant Tortoise Sanctuary (Changuu Island), Zanzibar Park, and Zanzibar Nungwi Zoo. This study investigated the occurrence of gastrointestinal parasite stages in faecal samples from captive Aldabra giant tortoises and compared the proportion of positive samples among the three zoological facilities. A total of 84 fresh faecal samples were examined using Fülleborn’s flotation and sedimentation techniques. Because individual tortoises could not always be reliably identified during sampling, repeated sampling of the same animal cannot be excluded. Therefore, the reported values represent sample-level proportions rather than individual-level prevalence. Oxyurid-type nematode eggs were detected only in samples collected from tortoises housed at Zanzibar Nungwi Zoo, whereas no gastrointestinal helminth eggs or larvae were detected using the applied coprological methods in samples from the Prison Island Giant Tortoise Sanctuary and Zanzibar Park. The overall proportion of positive faecal samples was 13.1% (11/84; 95% CI: 6.7–22.2%), while 42.3% (11/26; 95% CI: 23.4–63.1%) of samples from Zanzibar Nungwi Zoo were positive. Differences in the proportion of positive faecal samples were observed among the three zoological facilities. These findings provide baseline data on gastrointestinal parasites in captive Aldabra giant tortoises on Zanzibar Island and support future parasite surveillance, health monitoring, and management in zoological collections.

## Simple Summary

Aldabra giant tortoises (*Aldabrachelys gigantea*) are an iconic species of high conservation value, yet information on their gastrointestinal parasites in captive populations remains limited. This study presents the first systematic multisite survey of gastrointestinal parasites in captive Aldabra giant tortoises from three zoological facilities on Zanzibar Island, Tanzania. A total of 84 faecal samples were examined to assess gastrointestinal parasite occurrence in animals without reported clinical signs and compare findings among facilities. Oxyurid-type nematode eggs were detected only in faecal samples collected at Zanzibar Nungwi Zoo, whereas samples from the other two facilities were negative. These differences among facilities suggest that management- and husbandry-related factors may influence parasite occurrence and warrant further investigation. The findings provide baseline information on the gastrointestinal parasites of captive Aldabra giant tortoises in Zanzibar and support the importance of routine health monitoring and appropriate hygiene practices to promote animal welfare and conservation management.

## 1. Introduction

The Aldabra giant tortoise (*Aldabrachelys gigantea* Schweigger, 1812) is endemic to the Aldabra Atoll, Seychelles, and is currently listed as Vulnerable on the International Union for Conservation of Nature (IUCN) Red List of Threatened Species [1,2,3]. Conservation programmes initiated during the second half of the twentieth century have resulted in the establishment of numerous ex situ populations in zoological collections, rescue centres, and conservation facilities worldwide through captive breeding and translocation programmes [4,5,6]. These populations play an important role in species conservation, public education, and scientific research.

Maintaining healthy captive populations is an essential component of conservation programmes for giant tortoises. Husbandry-related factors, including enclosure hygiene, stocking density, environmental conditions, and chronic stress, may influence susceptibility to infectious and parasitic diseases [7,8]. Gastrointestinal parasitism is among the most frequently reported health concerns in captive chelonians and may adversely affect animal welfare, particularly under conditions that facilitate repeated environmental contamination and parasite transmission [9,10,11].

Oxyurid nematodes are the most frequently reported gastrointestinal helminths of herbivorous tortoises, commonly occurring as colonic parasites in both private and zoological collections, where they are most often detected in clinically healthy individuals [9,10,11,12,13,14,15,16]. At low infection intensities, oxyurids are generally considered to have low pathogenic potential and may form part of the normal intestinal fauna [10,16,17,18,19]. However, heavy parasite burdens have been associated with anorexia, weight loss, diarrhoea, poor body condition, growth disturbances, reduced fertility, increased susceptibility to secondary infections, intestinal obstruction, and, in hibernating species, post-hibernation mortality [9,20,21]. Besides oxyurids, tortoises may harbour a diverse gastrointestinal parasite fauna, including *Angusticaecum* spp., *Strongyloides* spp., and intestinal protozoa such as *Blastocystis*, *Hexamita*, *Entamoeba*, *Balantidium*, and *Nyctotherus* spp. [9,10,11,12,22].

Previous studies have shown that the occurrence and intensity of gastrointestinal parasite infections may differ between free-ranging and captive chelonians, reflecting differences in host density, environmental contamination, husbandry practices, and opportunities for reinfection [15,23,24]. These findings underline the importance of regular coprological surveillance in zoological collections [21].

Although gastrointestinal parasites have been reported in numerous chelonian species, dedicated investigations of gastrointestinal parasites in *Aldabrachelys gigantea* remain scarce. Most published studies involving this species have focused on conservation biology, genetics, nutrition, husbandry, or gut microbiota [2,5,23,25]. Available reports of gastrointestinal parasites are largely restricted to surveys involving multiple tortoise species, with only occasional records of oxyurid nematodes in *Aldabrachelys gigantea* [10,13]. Information from introduced captive populations maintained in East Africa is particularly limited.

Zanzibar Island, an autonomous part of the United Republic of Tanzania, hosts several zoological collections that maintain captive populations of *Aldabrachelys gigantea* under different management conditions. To our knowledge, no comparative coprological survey has investigated gastrointestinal parasites in captive Aldabra giant tortoises maintained in these zoological collections. Therefore, this study aimed to investigate the occurrence of gastrointestinal parasite stages in faecal samples collected from captive *Aldabrachelys gigantea* housed in three zoological collections on Zanzibar Island, Tanzania, using standard coprological techniques.

## 2. Materials and Methods

### 2.1. Study Population and Zoological Collections

A parasitological study was conducted in 2026 on captive Aldabra giant tortoises (*Aldabrachelys gigantea*) housed at three zoological and conservation facilities on Zanzibar Island, Tanzania: the Prison Island Giant Tortoise Sanctuary (Changuu Island), Zanzibar Nungwi Zoo (Baraka Natural Aquarium), and Zanzibar Park.

Fresh faecal samples were collected between 3 and 10 May 2026. Sampling was performed over four consecutive mornings at the Prison Island Giant Tortoise Sanctuary (3–6 May 2026), two consecutive mornings at Zanzibar Nungwi Zoo (7–8 May 2026), and two consecutive mornings at Zanzibar Park (9–10 May 2026).

A total of 84 fresh faecal samples were collected from apparently healthy captive Aldabra giant tortoises. Forty-four samples were collected at the Prison Island Giant Tortoise Sanctuary from tortoises estimated to be approximately 20–160 years old, 26 samples at Zanzibar Nungwi Zoo from tortoises approximately 20–80 years old, and 14 samples at Zanzibar Park from tortoises approximately 20–80 years old. Estimated ages were based on information provided by the facility staff and available animal records. Only clinically healthy tortoises were included in the study based on routine observations and information obtained from the facility staff. According to the zoological staff, the captive populations consisted of approximately 120 tortoises at the Prison Island Giant Tortoise Sanctuary, 35 tortoises at Zanzibar Nungwi Zoo, and 25 tortoises at Zanzibar Park. At the Prison Island Giant Tortoise Sanctuary, tortoises were maintained as a free-ranging population within a large open area without subdivision into enclosures. At Zanzibar Nungwi Zoo, approximately 35 tortoises were housed together in a single enclosure, whereas at Zanzibar Park approximately 25 tortoises were maintained in three enclosures containing about 8–9 animals each. Because individual tortoises could not always be reliably identified during sampling, the exact proportion of animals sampled from each population could not be determined, and repeated sampling of the same individual cannot be excluded. Information on previous anthelmintic treatments, quarantine procedures, and recent animal movements between institutions was not available.

### 2.2. Sample Collection

Fresh faecal samples were collected immediately after observed defecation, whenever possible, to minimize environmental contamination and the risk of cross-contamination between samples. Disposable gloves and sterile plastic spatulas were used for each collection. Approximately 100 g of faecal material was collected from each defecation event and placed into separate polyethylene zip-lock bags pre-labelled with the collection site, sample serial number, and date of collection. Each sample was assigned a unique serial number. However, because individual tortoises could not be reliably identified during every sampling event, repeated sampling of the same animal cannot be excluded.

Following collection, the samples were stored in portable cooling containers at 4–8 °C and transported to the laboratory for parasitological examination. Until examination, all samples were maintained under refrigerated conditions. The interval between sample collection and laboratory examination ranged from 24 to 96 h (median: 48 h).

### 2.3. Coprological Examination

Each faecal sample was examined using two conventional coprological techniques: Fülleborn’s flotation method and the simple sedimentation method.

For the flotation technique, approximately 5 g of faecal material was thoroughly homogenized with 20–30 mL of saturated sodium chloride (NaCl) solution (specific gravity 1.20). The suspension was filtered through gauze into a flotation vessel, and additional flotation solution was added until a convex meniscus was formed. The samples were left undisturbed for 30–60 min, allowing parasite eggs to float and concentrate at the surface. The surface layer was then collected using a wire loop, transferred onto a glass microscope slide, covered with a coverslip, and examined microscopically.

For the sedimentation technique, approximately 5 g of faecal material was homogenized with tap water, filtered through gauze into a 400 mL conical glass, and diluted with water to the full volume of the vessel. After sedimentation for 10–15 min, the supernatant was carefully decanted and replaced with fresh water. The washing and sedimentation procedure was repeated three to four times at approximately 5-min intervals until the supernatant became clear. After the final sedimentation, the supernatant was discarded, and the remaining sediment (approximately 4–5 mL) was transferred to a Petri dish for microscopic examination.

### 2.4. Microscopic Examination and Morphological Identification

For each faecal sample, three flotation preparations and one sedimentation preparation were examined. Flotation preparations mounted on glass microscope slides were examined using a ZEISS light microscope (Carl Zeiss Microscopy GmbH, Jena, Germany) at total magnifications of ×100 and ×200, whereas sedimentation preparations were examined directly in the Petri dish at a total magnification of ×100. The entire sediment contained in the Petri dish was systematically examined.

Parasite identification was performed by a single observer and was not independently reviewed by a second observer. The observer was aware of the zoological collection from which each sample originated (i.e., the examination was not performed under blinded conditions). Parasitic structures were identified based on their morphological characteristics, including size, shape, eggshell morphology, and internal contents, using published diagnostic keys and specialized parasitological literature for reptiles [26,27]. Parasite eggs were identified to the highest possible taxonomic level supported by the morphological features observed during coprological examination.

### 2.5. Statistical Analysis

Descriptive statistics were used to summarize the proportion of positive faecal samples in each zoological collection and for the complete sample set. Exact two-sided 95% confidence intervals (CIs) for the sample-level proportions were calculated using the Clopper–Pearson method in IBM SPSS Statistics for Windows, Version 32.0 (IBM Corp., Armonk, NY, USA). The calculation of these confidence intervals assumes independent observations. However, because individual tortoises could not always be reliably identified and repeated sampling of the same animal could not be excluded, this assumption could not be confirmed. Therefore, the reported confidence intervals should be interpreted with caution and as descriptive intervals for sample-level proportions rather than estimates of individual-level prevalence.

## 3. Results

During coprological examination using Fülleborn’s flotation and simple sedimentation methods, parasitic structures were detected exclusively in faecal samples collected from tortoises housed at Zanzibar Nungwi Zoo. No parasite eggs, larvae, or other diagnostic stages were detected in samples from the Prison Island Giant Tortoise Sanctuary or Zanzibar Park.

Of the 26 faecal samples collected from Zanzibar Nungwi Zoo, 11 were positive for oxyurid-type nematode eggs, corresponding to a sample-level proportion of positive faecal samples of 42.3% (95% CI: 23.4–63.1%). The remaining 15 faecal samples were negative. None of the 44 faecal samples from the Prison Island Giant Tortoise Sanctuary or the 14 faecal samples from Zanzibar Park contained detectable gastrointestinal parasite stages.

Overall, 11 of the 84 faecal samples were positive for oxyurid-type nematode eggs, corresponding to an overall sample-level proportion of positive faecal samples of 13.1% (95% CI: 6.7–22.2%) (Table 1).

Fülleborn’s flotation method detected oxyurid-type nematode eggs in all 11 positive samples, whereas the sedimentation method detected eggs in 4 of these samples. Four samples were positive by both methods, while seven samples were positive only by flotation. No samples were positive exclusively by sedimentation.

**Figure 1:**
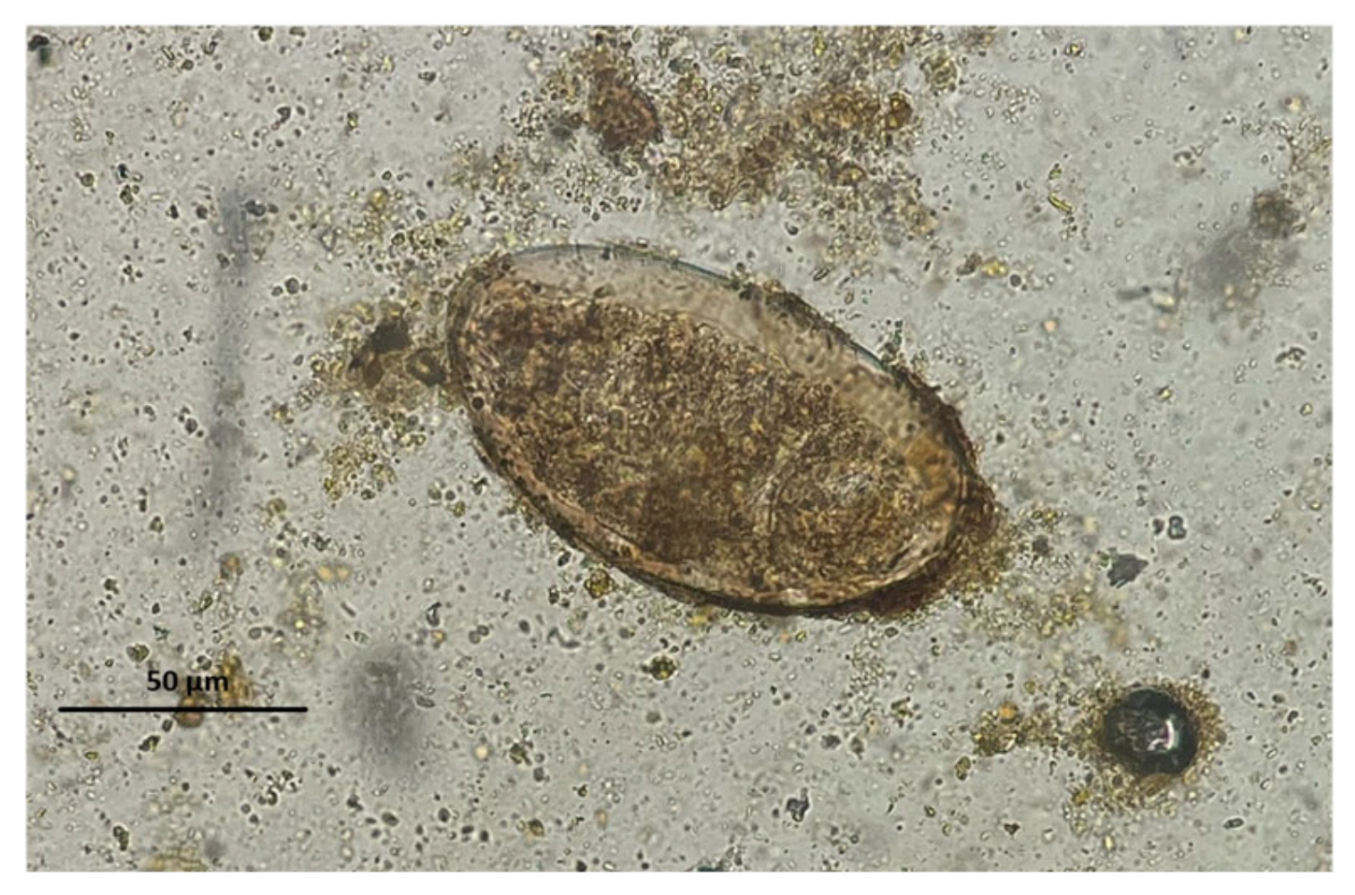
Oxyurid-type nematode egg recorded from faecal samples using the flotation method (×200).

**Figure 2:**
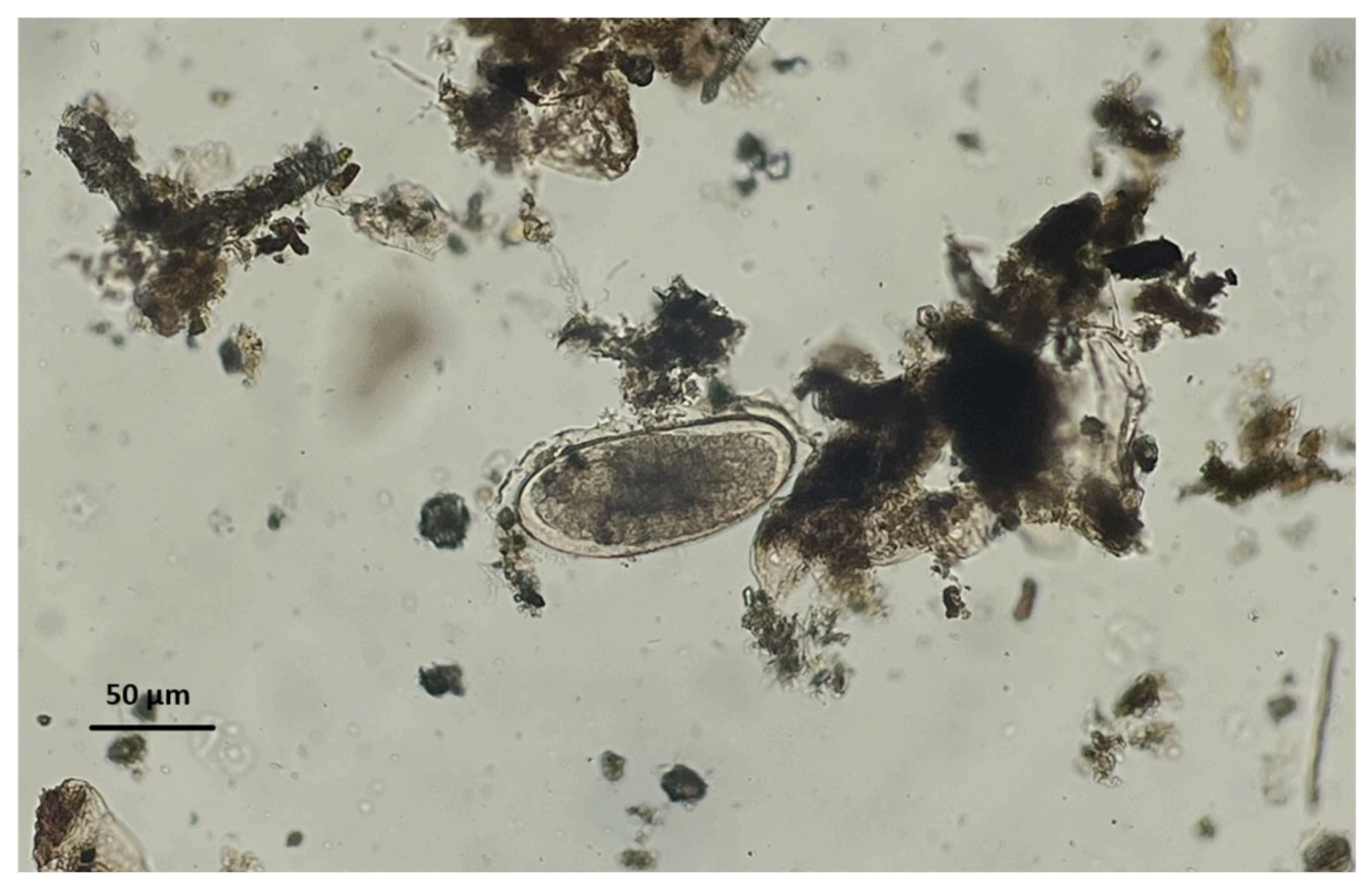
Oxyurid-type nematode egg recorded from faecal samples using the sedimentation method (×100).

Oxyurid-type nematode eggs were detected by both coprological methods and are shown in Figure 1 (flotation) and Figure 2 (sedimentation).

Morphologically, the detected eggs were elongated to ellipsoidal, with a smooth, thin shell, and distinct internal contents. Morphometric measurements were obtained from 10 representative eggs (*n* = 10). Egg length was 120.5 ± 9.8 µm (range: 100–130 µm), whereas egg width was 47.5 ± 5.9 µm (range: 40–55 µm). Based on their morphological characteristics and morphometric measurements, the eggs were identified as oxyurid-type nematode eggs.

## 4. Discussion

The present study provides the first systematic data on the occurrence of gastrointestinal parasites in captive *Aldabrachelys gigantea* from zoological collections on Zanzibar Island, Tanzania. Oxyurid-type nematode eggs were detected exclusively in faecal samples collected at Zanzibar Nungwi Zoo, suggesting that parasite occurrence may vary among geographically close captive populations maintained under different husbandry conditions.

The present findings are consistent with previous studies demonstrating that oxyurid nematodes are the most frequently detected gastrointestinal helminths of herbivorous tortoises in both captive and free-ranging populations [9,10,11,12,13,14,15,16]. At low infection intensity, they are generally regarded as part of the normal intestinal parasitofauna and can occur without pronounced clinical symptoms. However, under conditions of increased animal density, chronic stress, poor hygienic conditions, or compromised immune status, these parasites can reach high intensity and be associated with clinical manifestations such as anorexia, weight loss, diarrhoea, lethargy, and impairments in the general condition of the animals [20,21].

The observed differences among the three zoological collections may reflect variation in enclosure characteristics, group size, husbandry practices, veterinary management, or other environmental factors. One possible explanation for the exclusive detection of oxyurid-type eggs in Zanzibar Nungwi Zoo is that approximately 35 tortoises were maintained within a single enclosure, potentially increasing contact between individuals and facilitating environmental contamination and repeated exposure to infective stages. In contrast, tortoises at Zanzibar Park were distributed among three separate enclosures (approximately 8–9 animals per enclosure), whereas those at the Prison Island Giant Tortoise Sanctuary roamed freely within a large open area without enclosure subdivision. However, because enclosure size, hygiene practices, veterinary management, and other husbandry-related variables were not systematically evaluated, these interpretations remain hypothetical and require further investigation.

Several limitations should be considered when interpreting these findings. The study was based on qualitative coprological examination and therefore did not assess infection intensity by determining egg counts per unit of faecal material. Consequently, the relationship between parasite burden and clinical significance could not be evaluated. Furthermore, parasite identification relied on egg morphology supported by morphometric measurements and was not confirmed using molecular methods. Samples were collected opportunistically from freshly voided faeces, and individual tortoises could not always be reliably identified. Therefore, repeated sampling of the same individual could not be excluded, and the independence of observations could not be confirmed. Accordingly, the reported proportions and their confidence intervals should be interpreted as descriptive measures at the sample level rather than estimates of parasite occurrence among individual tortoises.

The observed variation in parasite occurrence among zoological collections highlights the importance of regular coprological monitoring, routine assessment of enclosure hygiene and sanitation, and clinical evaluation of animals with positive coprological results before treatment decisions are made. Such measures may improve parasite surveillance, reduce environmental contamination and reinfection, and contribute to maintaining optimal animal health and welfare.

From a conservation perspective, maintaining healthy ex situ populations is an essential component of long-term conservation programmes for *Aldabrachelys gigantea*. Regular parasitological surveillance may help reduce health risks associated with animal transfers between zoological institutions and future conservation translocations.

Although oxyurid infections have previously been reported in *Aldabrachelys gigantea* from other geographical regions [10,13], the present study represents the first systematic report on gastrointestinal parasites in captive *Aldabrachelys gigantea* from zoological collections on Zanzibar Island, Tanzania. These findings provide baseline data for future comparative and epidemiological studies of gastrointestinal parasites in captive giant tortoises.

## 5. Conclusions

The present study reports, for the first time, the occurrence of oxyurid-type nematode eggs in captive Aldabra giant tortoises (*Aldabrachelys gigantea*) from zoological collections on Zanzibar Island, Tanzania. The overall proportion of positive faecal samples (13.1%) and the differences observed among the three zoological collections highlight the value of routine parasitological monitoring in captive populations of this long-lived species.

As *Aldabrachelys gigantea* is currently classified as Vulnerable on the IUCN Red List of Threatened Species [3], routine parasitological monitoring may support veterinary management and conservation efforts for captive populations maintained in zoological institutions.

The findings provide regional baseline data on the gastrointestinal parasite status of captive *A. gigantea* on Zanzibar Island, contributing to the limited knowledge of gastrointestinal parasites in this species in East Africa. The reported proportions provide baseline information at the sample level and should not be interpreted as estimates of parasite occurrence among individual tortoises, as repeated sampling of the same individual could not be excluded. The present findings also highlight the need for future studies incorporating quantitative coprological, morphological, and molecular approaches to improve parasite identification and advance understanding of parasite epidemiology in captive giant tortoises.

## Figures and Tables

**Table 1:** Parasitological results from the studied populations of *Aldabrachelys gigantea* on Zanzibar Island.

Location	N	Positive Samples	Negative Samples	Proportion of Positive Samples (%)	95% CI	Parasite Stages Detected
Prison Island Giant Tortoise Sanctuary	44	0	44	0	0.0–8.0	Not detected
Zanzibar Park	14	0	14	0	0.0–23.2	Not detected
Zanzibar Nungwi Zoo	26	11	15	42.3	23.4–63.1	Oxyurid-type nematode eggs
Total	84	11	73	13.1	6.7–22.2	Oxyurid-type nematode eggs

Abbreviations: N, number of faecal samples examined; CI, 95% confidence interval. Exact two-sided 95% confidence intervals for sample-level proportions were calculated using the Clopper–Pearson method.

## Data Availability

The original data presented in this study are contained within the article and are available from the corresponding author upon reasonable request.

## References

[1] Rhodin A.G.J., Iverson J.B., Fritz U., Gallego-García N., Georges A., Shaffer H.B., Van Dijk P.P. (2025). Turtles of the World: Annotated Checklist and Atlas of Taxonomy, Synonymy, Distribution, and Conservation Status.

[2] Çilingir F.G., A’Bear L., Hansen D., Davis L.R., Bunbury N., Ozgul A., Croll D., Grossen C. (2022). Chromosome-level genome assembly for the Aldabra giant tortoise enables insights into the genetic health of a threatened population. Gigascience.

[3] Tortoise & Freshwater Turtle Specialist Group *Geochelone gigantea*. The IUCN Red List of Threatened Species 1996: E.T9010A12949962.

[4] Swingland I.R., Klemens M.W. (1989). The Conservation Biology of Tortoises.

[5] Spratt D.M.J. (1989). Operation Curieuse: A conservation programme for the Aldabra giant tortoise *Geochelone gigantea* in the Republic of Seychelles. Int. Zoo Yearb..

[6] Griffiths C.J., Zuël N., Tatayah V., Jones C.G., Griffiths O., Harris S. (2012). The Welfare Implications of Using Exotic Tortoises as Ecological Replacements. PLoS ONE.

[7] Bauer T., Reese S., Koelle P. (2019). Nutrition and husbandry conditions of Palearctic tortoises (*Testudo* spp.) in captivity. J. Appl. Anim. Welf. Sci..

[8] Pasmans F., Blahak S., Martel A., Pantchev N. (2008). Introducing reptiles into a captive collection: The role of the veterinarian. Vet. J..

[9] Nonnis F., Tamponi C., Pinna S., Diana F., Pudda F., Muzzeddu M., Cavallo L., Zeinoun P., Carta C., Varcasia A. (2024). Epidemiological survey of gastrointestinal helminths and protozoa in Testudines from Sardinia, Italy. Vet. Parasitol. Reg. Stud. Rep..

[10] Hallinger M.J., Taubert A., Hermosilla C., Mutschmann F. (2018). Occurrence of health-compromising protozoan and helminth infections in tortoises kept as pet animals in Germany. Parasites Vectors.

[11] Hedley J., Eatwell K., Shaw D.J. (2013). Paper: Gastrointestinal parasitic burdens in UK tortoises: A survey of tortoise owners and potential risk factors. Vet. Rec..

[12] Laghzaoui E.M., Amahmid O., Abbad A., El Mouden E.H. (2021). Prevalence and intensity of gastrointestinal parasites in the vulnerable spur-thighed tortoise (*Testudo graeca*) from the central-western of Morocco. Basic Appl. Herpetol..

[13] Tang P.K., Pellett S., Blake D.P., Hedley J. (2022). A prospective randomised clinical trial to compare the efficacy of Profender^®^ with Panacur^®^ against oxyurids in tortoises. Testudo.

[14] Dipineto L., Capasso M., Maurelli M.P., Russo T.P., Pepe P., Capone G., Fioretti A., Cringoli G., Rinaldi L. (2012). Survey of co-infection by *Salmonella* and oxyurids in tortoises. BMC Vet. Res..

[15] Chávarri M., Berriatua E., Giménez A., Gracia E., Martínez-Carrasco C., Ortiz J.M., de Ybánez R.R. (2012). Differences in helminth infections between captive and wild spur-thighed tortoises *Testudo graeca* in southern Spain: A potential risk of reintroductions of this species. Vet. Parasitol..

[16] Galosi L., Attili A.R., Perrucci S., Origgi F.C., Tambella A.M., Rossi G., Cuteri V., Napoleoni M., Mandolini N.A., Perugini G. (2021). Health assessment of wild speckled dwarf tortoises, *Chersobius signatus*. BMC Vet. Res..

[17] Traversa D., Capelli G., Iorio R., Bouamer S., Cameli A., Giangaspero A. (2005). Epidemiology and biology of nematodofauna affecting *Testudo hermanni*, *Testudo graeca* and *Testudo marginata* in Italy. Parasitol. Res..

[18] Walden H.D.S., Greiner E.C., Jacobson E.R., Jacobson E.R., Garner M.M. (2021). Parasites and parasitic diseases of reptiles. Infectious Diseases and Pathology of Reptiles: Color Atlas and Text.

[19] Canelas V.L.P., Santana R.L.S., Carvalho E.L., Giese E.G. (2023). *Ozolaimus megatyphlon* and *Ozolaimus cirratus* parasitizing *Iguana iguana* (Linnaeus, 1758) from Marajó Island, Pará, Brazil: New occurrence and morphological redescription. Rev. Bras. Parasitol. Vet..

[20] Sharun K., Satheesh A., Alexander J. (2020). Diagnosis and therapeutic management of gastrointestinal parasitism among the captive population of Indian star tortoise (*Geochelone elegans* Schoepff, 1795) at Zoological Garden, Thiruvananthapuram, Kerala, India. J. Parasit. Dis..

[21] Dantas D., Batista C.L., Castro M.J., Alvura N., Mateus T.L. (2025). Gastrointestinal Parasites in Reptiles from a Portuguese Zoo. J. Zool. Bot. Gard..

[22] Rataj A.V., Lindtner-Knific R., Vlahović K., Mavri U., Dovč A. (2011). Parasites in pet reptiles. Acta Vet. Scand..

[23] Sandri C., Correa F., Spiezio C., Trevisi P., Luise D., Modesto M., Remy S., Muzungaile M.M., Checcucci A., Zaborra C.A. (2020). Fecal Microbiota Characterization of Seychelles Giant Tortoises (*Aldabrachelys gigantea*) Living in Both Wild and Controlled Environments. Front. Microbiol..

[24] Silva A.M.D., Toqueto J., Carvalho N., Ramos D.G.S., Melo A.L.T. (2025). Gastrointestinal parasites of captive and free-ranging wild animals in the state of Mato Grosso do Sul. Rev. Bras. Parasitol. Vet..

[25] Zakaria D., Sandri C., Modesto M., Spiezio C., Scarafile D., Cedras A., Borruso L., Manghi P., Trevisi P., Segata N. (2025). Disentangling the gut microbiota of Aldabra giant tortoises of different ages and environments. PeerJ.

[26] Schneller P., Pantchev N. (2008). Parasitology in Snakes, Lizards and Chelonians—A Husbandry Guide.

[27] Petter A.J. (1961). Redescription et analyse critique de quelques especes d’Oxyures de la tortue grecque (*Testudo graeca* L.). Diversite des structures cephaliques. Ann. Parasitol. Hum. Comp..

